# Evaluation of Immunomodulatory and Hematologic Cell Outcome in Heroin/Opioid Addicts

**DOI:** 10.1155/2018/2036145

**Published:** 2018-12-09

**Authors:** Derya Guzel, Ahmet Bulent Yazici, Esra Yazici, Atila Erol

**Affiliations:** ^1^Department of Physiology, Faculty of Medicine, Sakarya University, 54100 Sakarya, Turkey; ^2^Department of Psychiatry, Faculty of Medicine, Sakarya University, 54100 Sakarya, Turkey

## Abstract

The long-term use of opioids leads alternations in both innate-adaptive immune systems and other diagnostic hematologic cells. The purpose of this study is to evaluate the alterations of these parameters in patients with heroin/opioid addictions. Adults, meeting the Fifth Edition of the Diagnostic and Statistical Manual of Mental Disorders (DSM-5) criteria of the American Psychiatric Association regarding opioid use disorder (Heroin Group or HG, n = 51) and healthy controls (Control Group or CG, n = 50), were included in the study. All hematological parameters, inflammation indexes (neutrophil-lymphocyte ratio and platelet-lymphocyte ratio), and iron panel were compared with the controls. Mean corpuscular volume, red blood cell distribution width, mean corpuscular hemoglobin content, unsaturated iron-binding capacity, and total iron-binding capacity were significantly higher in HG compared to CG, while red blood cell count, hemoglobin, hematocrit, and serum iron levels were significantly lower. Additionally, platelet and platelet distribution width were significantly high while mean platelet volume was low in HG. Regarding the parameters related to immunity, white blood cell, neutrophil count, and neutrophil percentage were significantly high while lymphocyte percentage and basophils count were significantly low. Besides, inflammatory indexes were significantly higher in HG compared to CG. Intravenous administration of heroin resulted in lower levels of hemoglobin, hematocrit, and mean corpuscular volume than inhalation and intranasal administration. Our data demonstrated that chronic use of opioids is related to all of the hematologic series. The chronic use of opioid alters the immunologic balance in favor of innate immunity cells and changes the hematometric/morphometric characteristics of erythrocytes. What is more, the route of heroin administration should be taken into consideration as well. This study may lead to a better understanding of the hematological effects of heroin/opioid use in patients with relevant addictions.

## 1. Introduction

Heroin is a powerful opioid analgesic and it is metabolized from the active form of morphine and a less active form of mono-acetyl morphine in 15-20 minutes. Due to the fact that it is more lipophilic, it passes the blood-brain barrier (BBB) more easily, and it is more potent than morphine [1]. The heroin may directly affect the immune system via the opiate receptors or indirectly through the nervous system. In addition to the known effect of the heroin on neuronal cell groups, in the studies conducted, it has been shown that, by activating tyrosine kinases in nonneuronal cell groups [2], it triggers inflammatory processes in the brain and facilitates the release of histamine that can directly affect the dopamine system [3–5]. In some case reports, eosinophilia was detected in heroin users [6]. It has also been shown that heroin also changes the amounts of major and trace element [7]. Among these elements, iron is known to have significance on tissue oxygenation as well as an effect on cognitive functions. Because of its importance in iron cell functions and in the course of the disease, the change in concentrations may disrupt the blood cell physiology, especially the erythrocyte series [8]. Thus, the systemic side effects of heroin are of interest to many studies on neuroimmunology and hematology.

The immune system is important for opioid users because the individuals with heroin use are in a high-risk group for the development of infectious diseases such as HIV and Hepatitis B and C as much as this addiction itself is a chronic disease causing numerous complications including alterations in immune responses [9, 10]. These functional alterations caused by opioids in immune cell functions have been investigated since the 1970s [11]. Effects of opioids on immune systems have been shown in in vivo and in vitro studies, and recent studies have shown that the effects of opioids on the immune system are more complex than it was previously considered [12]. One of the effects of opioids on the innate immune system occurs with the alterations in the leukocytes' functions. Leukocytes are a large family in the immune system, and they include mast cells, eosinophils, and basophils [11]. Meanwhile, changes in the activity of macrophage and interleukins were shown in the in vivo exposure of opioids [13]. In addition, the chronic exposure to the morphine has been found to be associated with the decreased lymphocytic series [14].

Chronic use of opioids alters the blood homeostasis via effects on the hematologic series. There are previous studies, which have shown that heroin addicts had significantly increased neutrophil count (NEUn), mean corpuscular volume (MCV), and hematocrit (HCT), while they had a significant reduction in lymphocytes (LYMn) and mean corpuscular hemoglobin concentration (MCHC) [5]. It is also observed that they have an increase in terms of mean platelet volume (MPV) [15]. Furthermore, changes in MCV, hemoglobin (HGB), HCT, red distribution width (RDW) occurred while decreases in red blood cell count (RBC) and increases in mean corpuscular hemoglobin content (MCH), MCHC, and RDW [16] were observed compared to the healthy individuals. Hematopoietic cells can be affected directly or indirectly due to the interaction with blood immune cells, and their morphological and functional alterations may occur.

The inflammatory indexes (neutrophil-lymphocyte ratio or NLR, platelet-lymphocyte ratio, or PLR) have been shown to be related to chronic diseases and psychiatric disorders in recent studies [17–19]. Neutrophils increase as a group of cells fighting inflammatory conditions. The amount of the second most common cell group of white blood cells, i.e., lymphocytes, relatively decreases since the inflammatory state is dominated by stress hormones. NLR, as an indicator of subclinical inflammation, increases the chances of various disorders such as coronary artery disease, malignancy, and autoimmune disease [20–24]; and also it has been shown to be associated with psychiatric disorders such as chronic stress [25] and schizophrenia [26]. In cases of systemic inflammation, thrombocytes increase even more due to the increased endothelial damage. Similar to NLR, PLR is also one of the indexes to determine the inflammatory balance [27].

The immunological and hematological effects of opioids have been investigated in various studies, yet, there have been no studies known on the inflammatory indexes of patients with heroin/opioid use disorder. The aim of the present study, therefore, is to assess the effects of heroin addiction on hematological factors, specifically considering the inflammatory indexes in heroin/opioid-addicted patients.

## 2. Method

### 2.1. Study Design

This is a cross-sectional study which was conducted at Alcohol and Substance Treatment and Training Centre inpatient clinic (SEAH-AMATEM) of Training and Research Hospital, Sakarya University, Turkey. The heroin/opioid-addicted group was constituted by the individuals, who have been using opioid and/or heroin for more than one year. Patients were interviewed by an experienced psychiatrist and were evaluated according to the DSM-5 criteria (opioid use disorders are not classified according to the opioid type in DSM-5) for heroin/opioid use disorder [28]. All patients included in the study were active heroin users according to their –positive- urine analysis results. Nonetheless, buprenorphine, methadone (not available in pharmacies in Turkey), meperidine (pethidine), morphine sulfate (not available in pharmacies in Turkey), and codeine users were not included into the study due to the urine analysis results for buprenorphine and anamnesis for others. The ones who have poly-substance use disorders or comorbid acute or chronic disorders (autoimmune or inflammatory diseases and neuropsychiatric disease) were excluded from the study as well. The control group consisted of voluntary individuals, who were healthy. Since the majority of the patients with the opioid use disorder were cigarette smokers, the control group was created by a matching number of smoking individuals, similarly. An approval was obtained from the Ethics Committee of Medical Faculty, Sakarya University.

### 2.2. Laboratory Analysis

#### 2.2.1. Serum Assays

On the first day of their hospitalization, ethylenediaminetetraacetic acid (EDTA) blood samples (BD Vacutainer K2EDTA Plus plastic tubes; Becton Dickinson, Franklin Lakes, NJ, USA) were collected from each patient at the time of admission and hemogram parameters were measured using aperture impedance technology. Moreover, the inflammation indexes (NLR and PLR) of the two groups were compared. NLR is the calculated ratio of the blood neutrophil to lymphocyte count, and PLR is calculated by dividing the absolute platelet count by the absolute lymphocyte count. Iron panels (serum iron, unsaturated iron capacity, and total iron capacity) were also measured. All hematological parameters, iron panel, and inflammatory indexes were compared to each other.

#### 2.2.2. Analysis of the Urine Tests

Cloned Enzyme Donor Immunoassay (CEDIA) was used as the immunoassay method during the analysis of the urine tests. The multiplex assay consists of multiple labels for the detection of amphetamine, benzodiazepine, barbiturate, opioids, MDMA (3,4-methylenedioxymethamphetamine, i.e., ecstasy), synthetic cannabinoids, tetrahydrocannabinol (THC), and buprenorphine for identifying the consumption of drugs; and ethyl glucuronide for the consumption of alcohol.

We used the cut-off levels in workplace drug tests, which are the recognized values for groups using drugs, to determine whether a sample is positive or negative for that group. Cut-off level for opioid is 300 ng/ml. Any result below the cut off is reported as negative, and results above the cut-off are reported as either nonnegative (for screening tests) or positive (for confirmed positive results). All of the patients had positive scores for 6 acetyl morphine and/or opioids in urine screening.

### 2.3. Statistical Analysis

Statistical Package for the Social Sciences (SPSS) version 22 for Windows (SPSS Inc., Chicago, IL, USA) was used for all statistical analyses. Quantitative data were expressed as Mean ± Standard Deviation (Mean ± Sd) for parametric analyses and Median ± Interquartile (Med ± IR) ranges for nonparametric analyses. Data were tested for normality, and the appropriate nonparametric or parametric statistics were used. The normality of distribution was checked initially by “One Sample Kolmogorov Smirnov Test”. Student's t-test was used to compare the linear variables and Levene's test was used for equality of the variances. Furthermore, Mann–Whitney U test was used to compare the linear variables which did not have the normal distribution. Any p-value less than 0.05 was accepted as significant.

### 2.4. Results General Characteristics and Clinical Outcomes of Patients

A total of 51 patients with heroin/opioid use disorder that comprise the heroin group (HG), and 50 healthy individuals that comprise the control group (CG) were involved in the study. Heroin group consisted of 49 men and 2 women, and the control group consisted of 48 men and 2 women. The average age of HG was 33.25 ± 8.79 years and the average age of CG was 30.84 ± 5.81; there was no statistically significant difference between the sexes as well as the ages of the groups.

### 2.5. The Comparison of Hematological Outcomes between HG and CG

**Figure 1:**
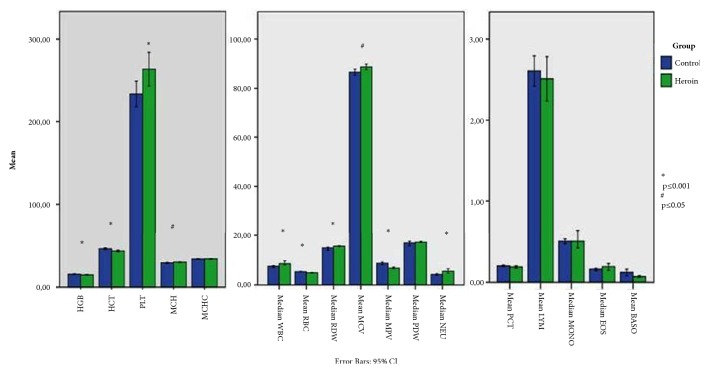
Mean and median count of hematological parameters.

The hematological parameters of HG and CG have been indicated as the mean and standard deviation values in Tables 1 and 2 and Figure 1. Erythrocyte series and serum iron profile characteristics of HG and CG are presented in Table 1. It can be seen that MCV (p<0.05), RDW (p<0.001), MCH (p<0.05), unsaturated iron-binding capacity (UIBC, p<0.001), and total iron-binding capacity (TIBC, p<0.05) were higher in HG compared to CG; and RBC (p<0.001), HGB (p=0.001), HCT (p<0.001), and serum iron (SI, p<0.001) were lower for the same comparison.

Parameters related to immune system cells and platelets of both groups are presented in Table 2. According to these numbers, WBC (p=0.001), NEU, and NEU% values were high (p<0.001), while LYM% (p<0.001) values were low. Again, PLT value was high (p<0.05), while MPV was low (p<0.001). Furthermore, inflammatory indexes were significantly higher in HG compared to CG (PLR, p<0.05 and NLR p<0.001).

### 2.6. Comparison of the Route of Administration

**Figure 2:**
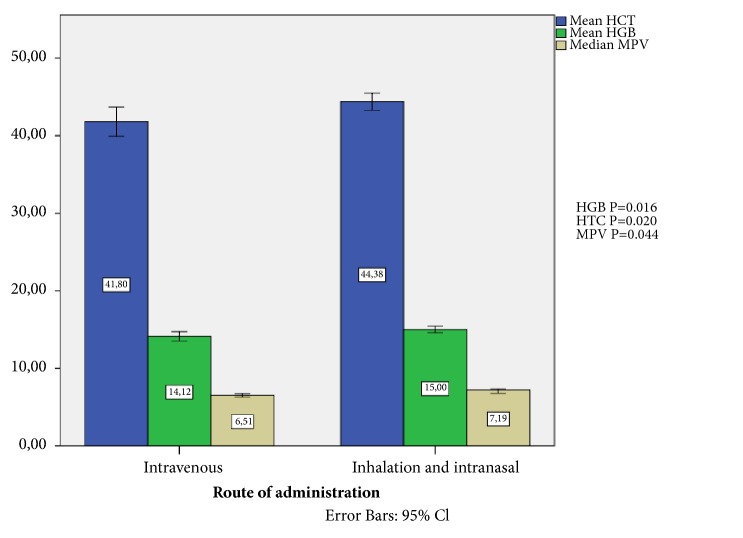
Comparison of the route of administration.

Among the participants, 23 patients were using the intravenous route, while 20 were using inhalation route, and only 8 were using intranasal route. We divided patients into two groups according to the route of administration as of* intravenous* and* other* (i.e., intranasal and inhalation). When comparing two groups according to hematological parameters it is seen that HGB, HTC, and MPV are lower in the* intravenous* administration group than the* other* group (Figure 2).

## 3. Discussion

In this study, hematological parameters of heroin addicts and healthy individuals in the control group who have similar general characteristics have been compared and several major results were obtained. Firstly, it was seen that erythrocyte series of the opioid users has declined. Secondly, while the numbers of platelets increase, their volume also decreases. Thirdly, it was also observed that there is a remarkable increase in inflammatory indexes with a significant increase in neutrophil in leucocytes.

Differences in the erythrocyte series occurred as giving higher MCV values and lower HGB, RBC, and HCT values. Moreover, their level of iron value is low and iron-binding capacity is high in comparison with the control group, which can be considered that this change depends on iron deficiency anemia. On the other hand, high MCV value in iron deficiency anemia, contrary to expectations, is associated with the problems related to nutrition such as vitamin B12 and folic acid deficiency [29]. This situation brings questions about the effects of probable malnutrition, which in fact have been overlooked in heroin addicts. In the current study, food regimen and nutritional status were not specifically assessed yet malnutrition due to different lifestyles in heroin addicts can be regarded as one of the important clinical problems of this study. Malnutrition problems of this patient group should be considered by the treatment establishments. In addition, since the increase in MCV has been considered as an inflammatory indicator [30], it has reminded that inflammatory processes should be reviewed for heroin patients. In this study, in addition to the MCV values, inflammatory indexes have also been evaluated. Consequently, NLR and PLR, which have been known as inflammatory indexes, have been found significantly high compared to the control group.

It is well known that the circulating WBC classification is exposed to relative changes when systemic inflammation occurs, these changes are typically represented by lymphopenia and neutrophilia [31] and finally affect the rates of NLR and PLR as proinflammatory indexes [32]. In the present study, it is determined that both of the higher platelet count and the lower lymphocyte count are the reasons of higher levels of PLR, and again, both higher levels in neutrophil count and lower levels of lymphocyte are the reasons for higher levels of NLR.

Opioids are widely used for the management of pain and the mechanisms related to pain are one of the common researched scientific topics about opioid users. Pain facilitation (hyperalgesia) has been the subject of research for many years. One of the known mechanisms of hyperalgesia is the activation of peripheral nerves in the brain or spinal cord dorsal horn after the release of soluble signaling molecules of immune cells stimulates signals of the formation of hyperalgesia [33]. In our study, it was shown that inflammatory responses were triggered in heroin users compared to control. It has been shown in the literature that chronic opioid use increases the secretion of cytokines and chemokines via *μ*-R. Previous studies have demonstrated that following *μ*-R-mediated inflammatory marker release neuroimmune activation changes with influencing glial cells and this is one of the mechanisms of opiate tolerance/hyperalgesia [34]. The released inflammatory molecules affect pre- or postsynaptic neurons or glial cells in order to induce opiate tolerance/hyperalgesia. [35] A limitation of the current study is that no hyperalgesia/tolerance was assessed; still, our results present data about the inflammatory mechanisms that may help to understand changes in pain response and this part needs further investigation.

In the present study, another result, which can be interpreted as a marker related to inflammatory processes, is the changes in the platelets. MPV has been shown to be a useful marker in the assessment of systemic inflammation and has been investigated in various inflammatory diseases, which also indicates disease activity [36, 37]. MPV is accepted as a manifestation of both proinflammatory and prothrombotic conditions. Large and small-sized circulating platelets may show the intensity of systemic inflammation. The intensive inflammatory conditions (e.g., active Rheumatoid Arthritis or FMF attacks) are associated with circulation of predominantly smaller platelets, whereas the same disorders during remission period and when they were controlled by anti-inflammatory drugs are associated with large circulating platelets [37]. Intensive inflammation comes with a decrease in the MPV autoimmune inflammatory diseases, probably because of the increased expenditure of platelets [38, 39]. In this study, while MPV values of heroin users were low, the numbers of platelets were high in the control group. This result may suggest an increased consumption of platelet and secondarily induced platelet production [37]. In the current study, decreased MPV and increased platelet count have been caused considering a proinflammatory and prothrombotic process.

One of the strengths of this study is that the route of administration of heroin/opiate has been evaluated as well. A significant difference was found between the groups, but there might be many reasons for this. The decrease in HGB and HTC values in patients using intravenous route may be caused by the bleeding during chronic use. In a study, white matter loss and hemoglobin and mean platelet volume decrease were found to be correlated with each other as a symptom of microvascular insufficiency and tissue hypoxia in sickle cell disease patients [40]. The assessment of similar findings in this patient group with a lack of a known direct explanation could create new research objectives.

Results of this study have revealed that there is an increase in inflammatory indexes of heroin addicts. However, previous studies stated that opioids have an immunosuppressive effect and lead to an exacerbation of immunological vulnerability in opioid users [41]. This ambivalence may be related to too many factors. Firstly, the heroin users in the current study are all chronic users who have an addiction for at least 1 year; therefore, all of them were long-term users. Chronic use of opioids may have different results than short-term users [42, 43]. The duration of exposure to the opioids leads to different results. Another reason for this opposing result might be the difference of evaluated parameters in previous studies as well as this one. One research has shown increased chemotactic activity of macrophage, decreased IL-1, TNF-*α* production, and chemokinesis that occur in in vivo opioids exposure [11]. Besides, exposure to morphine has been related to the decreased lymphocytic series and their precursors by decreased thymic and splenic weight and size [13]. These results have verified that immunologic balance has changed in the favor of innate immunity cells, and thus, it explains our inflammatory index changes. This is the first study that evaluated NLR and PLR as inflammatory indexes, while the previous studies were generally interested in hematological cell counts as well as volumes and functionality of macrophages [43]. Moreover, previous researches checked immunosuppression or immunomodulation parameters instead of the inflammatory response [43, 44]. Hence, it seems that there is ambivalence in the literature; however, when it is considered in detail it is understood that we did not completely evaluate the same issue either. That is to say, when the parameters are compared one by one, studies, which have results similar to this study, might be seen. For example, a previous study, which was conducted by Haghpanah and Afarinesh [5], has shown that heroin dependents had significantly increased neutrophil counts and MCV as well as a significant reduction of lymphocytes count. In addition, another study has also shown that there are increased MPV values in heroin addicts [15]. These parameters may be considered as the case of increasing inflammatory process rather than the immunosuppression.

Nonetheless, the current study also has several limitations. First of all, this not a prospective study, where the parameters were evaluated in a cross-sectional way by comparing with the control group. The study group consists of a small female sample size because heroin addiction is rarely seen in women in Turkey. Not being able to make a comparison between male and female in this study is also a limitation of the study. It has been hard to find initial hematological parameters of heroin addicts before the onset of their heroin use. It is well known that street heroin (illegal heroin) have a little active substance and large quantities of other chemical compounds [45]. Thus it might affect and change the immunological reactions of the substance. This can be counted as another limitation of this research. Furthermore, another limitation is the lack of data about vitamin levels of the patients.

On the other hand, this study has strong results because it has diagnosed the heroin users both with clinical evaluation and laboratory urine screening tests. In addition, differently from the previous studies in this area, both of the groups were smokers, so the probable confounding effect of smoking was eliminated.

The final results of this study might be expressed as hematological changes claimed malnutrition, and changes in the inflammatory indexes claimed inflammatory induction in heroin users. New studies including replication of this study and adding other inflammatory parameters will be beneficial for a better understanding of the subject.

## 4. Conclusion

Our findings show the negative effects of chronic opiate use on hematometric/morphometric characteristics of blood cells. Opiate users have an inflammatory condition compared to the control group. Inflammatory indexes and innate immunity cells are significantly increased in the heroin group in comparison to the control groups. These allostatic changes in the immune system of heroin addicts suggest scanning and researching these patients for immune system related to other medical results and disorders. Intravenous administration of heroin was related to lower levels of HGB, HTC, and MPV than inhalation and intranasal administration. As a result, it can be said that the route of administration of heroin should be taken into consideration in the relevant studies and in routine clinical assessments.

In addition, threshold/subclinical megaloblastic anemia with iron deficiency should be considered while the treatment of these patients is being planned. Discovering the impacts of opioids on hematological parameters may be beneficial for defining the homeostasis condition of the body, which leads to indications for treatment approaches.

## Figures and Tables

**Table 1:** Erythrocyte series and serum iron profile parameters of groups.

**Laboratory parameters**	**Statistic** ^**+**^	**HG (n=51)**	**CG (n=50)**	**d** **f** ^***π***^	**p-value**
Red Blood Cell Count (RBC, M/uL)	Mean ± Sd	4.88±0.49	5.32±0.43	97.97	**≤0.001** ^**#**^
Hemoglobin (HGB, g/dl)	Mean ± Sd	14.60±1.28	15.45±1.26	98.98	**0.001** ^**#**^
Hematocrit (HCT, %)	Mean ± Sd	43.21±3.80	45.95±3.25	97.18	**≤0.001** ^**#**^
Mean Corpuscular Hemoglobin (MCH, pg)	Mean ± Sd	29.98±1.45	29.08±1.96	90.12	**0.010** **∗**
Mean Corpuscular Hemoglobin Concentration (MCHC, g/dl)	Mean ± Sd	33.81±1.16	33.57±0.94	95.56	0.257
Red Cell Distribution Width (RDW, %)	Med ± IR	15.60±1.20	14.90±1.97	95.65	**≤0.001** ^**#**^
Mean Corpuscular Volume (MCV, fl)	Med ± IR	88.80±4.90	86.90±3.95	97.36	**0.013** **∗**
Serum Iron (SI, *μ*g/dL)	Mean ± Sd	66.00±28.11	102.44±35.80	92.89	**≤0.001** ^**#**^
Unsaturated Iron Binding Capacity (UIBC, *μ*g/dL)	Mean ± Sd	246.08±58.42	188.17±58.45	98.95	**≤0.001** ^**#**^
Total Iron Binding Capacity (TIBC, *μ*g/dL)	Mean ± Sd	311.92±49.16	292.11±43.74	98.08	**0.035** **∗**

*∗*Significant at the 0.05 level (two-tailed). ^#^Significant at the 0.001 level (two-tailed). ^+^Mean ± Standard Division (Mean ± SD) for parametric analysis and Median ± Interquartile (Med ± IR) range for nonparametric analysis. ^*π*^Welch-correction to the degrees of freedom (df).

**Table 2:** Inflammatory series and platelet parameters of both groups.

**Laboratory parameters**	**Statistic** ^**+**^	**HG (n=51)**	**CG (n=50)**	**d** **f** ^***π***^	**p-value**
Platelet (PLT, K/uL)	Mean ± Sd	263.73±72.22	233.90±54.30	92.80	**0.010** **∗**
Mean Platelet Volume (MPV, fl)	Med ± IR	6.77±1.08	8.72±2.10	79.44	**≤0.001** ^**#**^
Plateletcrit (PCT, GSD)	Mean ± Sd	0.19±0.05	0.20±0.04	93.18	0.154
Platelet Distribution Width (PDW, %)	Med ± IR	17.20±1.00	16.90±5.27	57.79	0.144
White Blood Cell Count (WBC, K/uL)	Med ± IR	8.55±3.59	7.50±1.68	75.48	**0.001** ^**#**^
Neutrophils (NEUn, K/uL)	Med ± IR	5.52±2.89	4.19±1.35	79.57	**≤0.001** ^**#**^
Neutrophil (NEU%, K/uL)	Mean ± Sd	63.50±7.60	56.47±7.56	98.97	**≤0.001** ^**#**^
Lymphocytes (LYMn, K/uL)	Mean ± Sd	2.51±0.96	2.60±0.65	88.09	0.558
Lymphocyte (LYM%, K/uL)	Mean ± Sd	27.22±7.16	34.88±7.09	98.98	**≤0.001** ^**#**^
Monocytes (MONOn, K/uL)	Med ± IR	0.50±0.33	0.50±0.18	81.14	0.311
Monocytes (MONO %, K/uL)	Mean ± Sd	6.18±1.76	6.35±1.74	98.98	0.624
Eosinophils (EOSn, K/uL)	Med ± IR	0.19±0.19	0.16±0.10	72.75	0.250
Eosinophils (EOS%, K/uL)	Med ± IR	2.11±1.81	1.88±1.33	86.38	0.696
Basophils (BASOn, K/uL)	Mean ± Sd	0.067±0.06	0.068±0.06	55.16	0.550
Basophils (BASO%, K/uL)	Mean ± Sd	0.77±0.34	0.84±0.47	89.12	0.421
Platelet / lymphocyte ratio (PLR)	Med ± IR	107.88±80.31	91.16±32.67	81.82	**0.014** **∗**
Neutrophil / lymphocyte ratio (NLR)	Med ± IR	2.31±1.42	1.61±0.95	78.36	**≤0.001** ^**#**^

*∗*Significant at the 0.05 level (two-tailed). ^#^Significant at the 0.001 level (two-tailed). ^+^Mean ± Standard Division (Mean ± SD) for parametric analysis and Median ± Interquartile (Med ± IR) range for nonparametric analysis. ^*π*^Welch-correction to the degrees of freedom (df).

## Data Availability

The data including names of the patients used to support the findings of this study are restricted by the Sakarya University Ethics Committee in order to protect patient privacy. Data are available from Dr. Derya GUZEL (deryaguzel@sakarya.edu.tr) for researchers who meet the criteria for access to confidential data.
